# Nutrient supply controls particulate elemental concentrations and ratios in the low latitude eastern Indian Ocean

**DOI:** 10.1038/s41467-018-06892-w

**Published:** 2018-11-19

**Authors:** Catherine A. Garcia, Steven E. Baer, Nathan S. Garcia, Sara Rauschenberg, Benjamin S. Twining, Michael W. Lomas, Adam C. Martiny

**Affiliations:** 1 Department of Earth System Science, University of California at Irvine, Irvine, CA 92617 USA; 2 Bigelow Laboratory for Ocean Sciences, East Boothbay, ME 04544 USA; 3 Department of Ecology and Evolution, University of California at Irvine, Irvine, CA 92617 USA; 4 Present Address: Maine Maritime Academy, Castine, ME, 04420 USA

## Abstract

Variation in ocean C:N:P of particulate organic matter (POM) has led to competing hypotheses for the underlying drivers. Each hypothesis predicts C:N:P equally well due to regional co-variance in environmental conditions and biodiversity. The Indian Ocean offers a unique positive temperature and nutrient supply relationship to test these hypotheses. Here we show how elemental concentrations and ratios vary over daily and regional scales. POM concentrations were lowest in the southern gyre, elevated across the equator, and peaked in the Bay of Bengal. Elemental ratios were highest in the gyre, but approached Redfield proportions northwards. As *Prochlorococcus* dominated the phytoplankton community, biodiversity changes could not explain the elemental variation. Instead, our data supports the nutrient supply hypothesis. Finally, gyre dissolved iron concentrations suggest extensive iron stress, leading to depressed ratios compared to other gyres. We propose a model whereby differences in iron supply and N_2_-fixation influence C:N:P levels across ocean gyres.

## Abstract (web summary)

The Indian Ocean provides a unique environmental gradient to test underlying drivers of the elemental composition of particulate organic matter. Here the authors show that nutrient supply, over temperature and biodiversity changes, controls regional variation of elemental ratios in the tropical Indian Ocean.

## Introduction

Elemental ratios of particulate organic matter (POM) are key to linking biogeochemical cycles. The carbon:nitrogen:phosphorus (C:N:P) ratio is often assumed globally constant at Redfield proportions (106C:16N:1P). However, recent observations show high ratios in nutrient-poor subtropical gyres and low ratios in nutrient-rich environments^1,2^. There are also ocean basin differences with higher C:P and N:P values in the North Atlantic subtropical gyre and lower ratios in other subtropical gyres^3,4^. However, many regions remain woefully under-sampled, especially in regards to particulate organic phosphorus (POP).

Individual studies have presented competing hypotheses explaining global variation in the elemental ratios of POM^5^. First, the translation-compensation hypothesis predicts a negative relationship between temperature and the cellular concentration of P-rich ribosomes as higher temperatures increase ribosomal translation efficiency^6^. This would lead to a positive relationship between temperature and C(N):P ratios. Second, the nutrient supply hypothesis predicts that nutrient stressed cells are frugal and have low cell quotas of the limiting nutrient. For example, this hypothesis predicts a negative correlation between C:P and ambient P availability^7,8^. Thirdly, the allometric diversity hypothesis predicts that smaller, nutrient uptake specialists like *Prochlorococcus* have elevated C:P and N:P in comparison to larger lineages like diatoms^1,8,9^. However, it is a challenge to separate these hypotheses as temperature, nutrient supply, and community composition strongly co-vary in the ocean.

The Indian Ocean (IO) accounts for 15–20% of global ocean net primary production^10^, but there are few published data that describe the ocean biogeochemistry of POM in this region. In the Indian Ocean spring inter-monsoon season, sea surface temperatures (SST) and macronutrient concentrations increase northwards. Strong gradients in temperature and nutrient concentrations in the surface ocean suggest three distinct regions: an oligotrophic, cooler (20.5–29.7 °C) Southern Indian Ocean gyre (SIO gyre); a warm (30.3–31.5 °C) upwelling region north of 10°S (EqIO); and a warm (29.1–32.6 °C), higher biomass region in the Bay of Bengal (BoB)^11^. Although surface nutrient concentrations are consumed to near depletion throughout the basin, two overturning thermohaline cells deliver nutrient-replete water close to the surface around 10°S and slightly north of the equator^12^. However, the northern branch of the cross-equatorial cell is not well defined^13^. The BoB also has elevated nutrient supply driven seasonally by coastal upwelling and river inputs, thereby leading to periods of increased productivity^14^. Thus, it appears that the warmest regions are also the most nutrient replete in the eastern Indian Ocean leading to temperature and macronutrient supply being uniquely positively correlated. As such, this region enables a test of our hypotheses for how phytoplankton stoichiometry ratios are controlled.

Here, we ask the following three questions about environmental and biological controls of biogeochemistry in the eastern Indian Ocean: How do POM concentrations and elemental ratios vary between regions and on short-term scales within regions? How do the phytoplankton community composition and environmental conditions relate to variation in POM concentrations and elemental ratios? Is the SIO gyre unique in terms of its POM concentrations, ratios, and controls compared to other oligotrophic gyres?

Our results suggest that nutrient supply is the leading driver of regional variation in elemental composition in the eastern Indian Ocean as well as other low latitude regions. However, the C:P ratio in the SIO gyre is low in comparison to other subtropical gyres leading us to propose that iron stress controls the POM C:P ratio in oligotrophic regions via regulation of N_2_-fixation. Thus, the unique biogeochemistry of the Indian Ocean provides key information for understanding the controls of ocean C:N:P.

## Results

### I09N transect environmental gradients

**Fig. 1:**
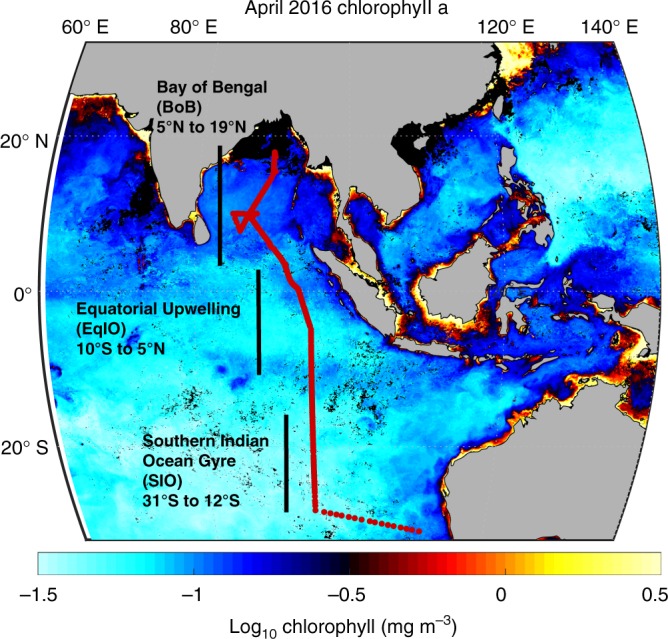
Study region. The transect sampling locations for GO-SHIP cruise IO9N are marked by the red dots. The approximate latitudinal range of proposed regions is marked by black bars. Chlorophyll concentrations are from MODIS-Aqua 4 km April 2016 monthly average^17^. Figure was created in MATLAB using fireice.m colormap package

**Fig. 2:**
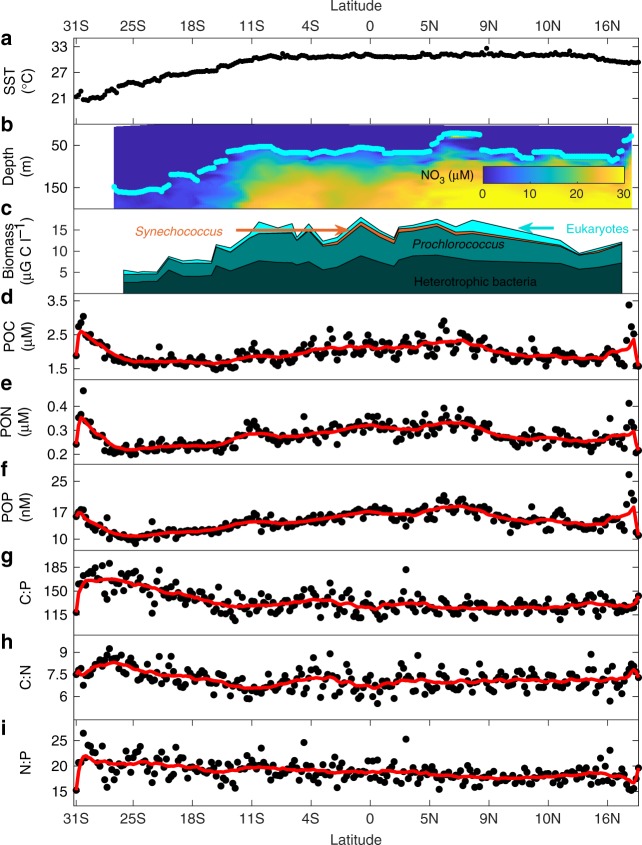
Observations of environmental conditions, relative phytoplankton biomass, POM concentrations, and ratios across the eastern tropical Indian Ocean. **a** Sea surface temperatures; **b** nitrate concentrations as shaded background and nutricline depth (depth with 1 µM [NO_3_]) marked by light blue dots; **c** Phytoplankton absolute biomass; **d** particulate organic carbon (POC); **e** particulate organic nitrogen (PON); **f** particulate organic phosphorus (POP); **g** POC:POP (C:P); **h** POC:PON (C:N); and **i** PON:POP (N:P). In panels **d**–**i**, averages of analytical triplicates are marked by black dots, the red line represents an 8-sample moving average, and elemental ratios are molar. First and last 4 end points are averaged over fewer than 8 points. For bacteria and phytoplankton: Bact(dark green) = Heterotrophic bacteria, Pro(greenblue) = *Prochlorococcus*, Syn(orange) = *Synechococcus*, and Euks(cyan) = Eukaryotes

To quantify the link between environmental gradients, phytoplankton community composition, POM concentrations, and ratios, we collected samples across 238 stations in the eastern Indian Ocean (Fig. 1, Supplementary Data 1). Both the western and eastern Indian Ocean experienced anomalously warm SST during the sampling period. However, there was an overall positive Indian Ocean Dipole (IOP +0.34, April 2016) since the eastern basin was cooler. These conditions favor wind patterns that promote upwelling off Indonesia^15^. Based on near surface temperature and nutrient concentration gradients, we classified the transect into the SIO gyre (31°S–12°S), an equatorial upwelling region (EqIO, 10°S–5°N), and the BoB (5°N–19°N) (Fig. 2a, b and Supplementary Fig. 1). Due to uncertainty in the SIO gyre-Indonesian through flow transition, 12°S was used instead of 10°S as the gyre northern cutoff. We used the depth of the 1 µM NO_3_ isocline to define the nutricline and applied this as a proxy for nutrient supply into the surface layer (Fig. 2b, Supplementary Fig. 2)^16^. SIO gyre had the lowest surface temperature and the deepest nutricline depth along the transect (129 m) (Supplementary Table 1 and Fig. 2a, b). EqIO was characterized by temperatures above 29 °C and the nutricline shoaled to 62 m. Increased nitrate concentrations below the nutricline near 10°S, the equator, and 5°N corresponded to bands of elevated chlorophyll (Fig. 1)^17^. This suggests high nutrient supply at these bands. In BoB, temperature was on average 30.8 °C, and the nutricline remained constant (61 m). Overall, there was a coupled negative relationship between SST and nutricline depth (*R* = −0.88). Thus, there were significant regional environmental differences, but in support of our prediction, a uniquely positive relationship between temperature and the nutrient supply.

### Short term and regional variation in POM

**Fig. 3:**
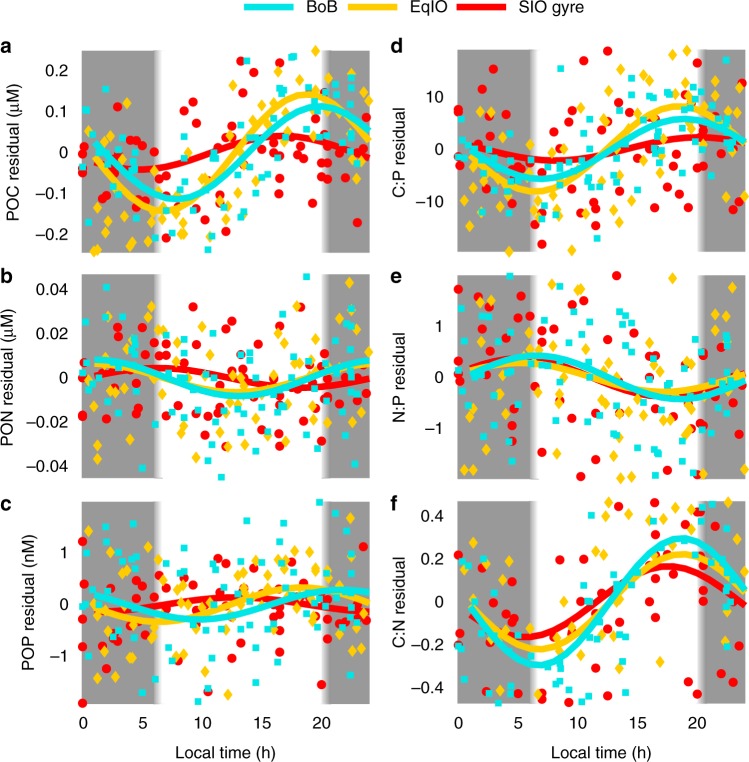
Diel variation in POM concentrations and ratios. Residuals are calculated as follows: data points subtracted from the 8-point smoothed line for **a** particulate organic carbon (POC), **b** particulate organic nitrogen (PON), **c** particulate organic phosphorus (POP), **d** POC:POP (C:P), **e** PON:POP (N:P), and **f** POC:PON (C:N). Points were plotted and a sine curve was fitted for each region. Bay of Bengal (BoB) is in cyan. Equatorial Upwelling (EqIO) is in gold. Southern Indian Ocean gyre (SIO gyre) is in red. The grey bar represents local nighttime and white bar represents daytime

We identified significant diel variability of POM concentrations and elemental ratios (Fig. 3). Particulate organic carbon (POC) had the strongest cycle with a maximum at dusk, and minimum at dawn. POP had a similar cycle, whereas particulate organic nitrogen (PON) cycled with a peak between midnight and 07:00 local time (Supplementary Table 1). The stronger oscillation in POC led to C:N and C:P maxima near dusk. The temporal shift in the peak of PON relative to POP led to a weak N:P ratio maximum at 17:00 local time. Over the course of a day, on average the ratio of C:P changed by 13.4, N:P by 0.64, and C:N by 0.58 in the eastern Indian Ocean. These daily ranges were comparable to differences observed between regions (Supplementary Table 1).

We next found distinct diel amplitudes across regions (Fig. 3). The smallest amplitudes for all POM concentrations and elemental ratios were observed in SIO gyre, but no significant differences between the BoB or EqIO regions (Supplementary Table 1). Using the daily POC range as a proxy for daily biomass accumulation, the highest normalized accumulation rates were observed on the coastal margin of Western Australia at 30.7°S, EqIO at 2.4°S, and intermittently northwards at 5°N, 8.5°N, and 17.2°N (Supplementary Fig. 3). In contrast, the POC normalized accumulation rates were dampened in SIO gyre. The nutrient and hydrography profiles suggested upwelling at ~4–8°N, where two of the POC normalized accumulation peaks were observed (Supplementary Fig. 1). Thus, there appeared to be increased daily carbon accumulation in regions with elevated nutrient availability.

**Fig. 4:**
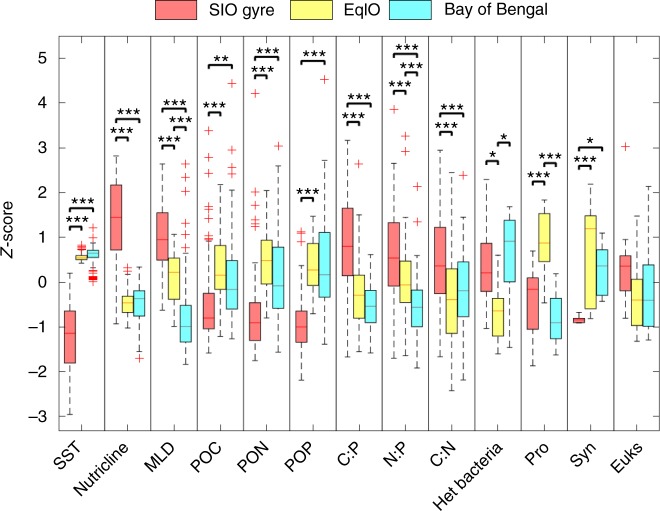
Regional variation in environmental parameters, relative biomass, and POM concentrations and ratios. Significant regional variation between specific regions is indicated by the number of stars (ANOVA, *p < 0.5, **p < 0.01, ***p < 0.001). For relative biomass; Het bacteria = heterotrophic bacteria, Pro = *Prochlorococcus*, Syn = *Synechococcus*, and Euks = Eukaryotes

We found significant regional variation in the concentration and ratios of POC, PON, and POP (Fig. 4 and Supplementary Table 2). POM concentrations were lowest in SIO gyre and higher northwards (Supplementary Table 1). In BoB, the POM concentrations decreased from 9°N to 15°N followed by a sharp increase in waters overlying the continental shelf (Fig. 2d–f). Although the nutricline shoals, nutrients may be entrained below the thermocline due to strong salinity gradients in BoB leading to low POM concentrations^18^. The elemental ratios followed similar declining northward trends, with high ratios in the SIO gyre and low ratios in the north. C:P and C:N decreased sharply during the transition from SIO Gyre (C:P 150, C:N 7.6) to EqIO (C:P 131, C:N 7.0), but stayed slightly above Redfield proportions in the EqIO and the BoB (C:P 127, C:N 7.1) (Fig. 2g–i). N:P decreased gradually northward throughout the transect (N:P: SIO gyre = 20.1 EqIO = 19.0, BoB = 17.9). Thus, there were clear regional differences in elemental ratios across the eastern Indian Ocean.

### Testing ecological stoichiometry hypotheses

We next tested the three proposed stoichiometry models for POM stoichiometry trends in the Indian Ocean. First, we addressed the allometric diversity hypothesis. Consistent with past studies^19^, *Prochlorococcus* dominated the phytoplankton portion with only small contributions from picoeukaryotic phytoplankton and *Synechococcus*. The picoeukaryotic phytoplankton increased in biomass north and south of the equator, while the *Synechococcus* biomass increase centered on the equator. Residual effects of coastal upwelling could explain the increases in *Synechococcus* in the EqIO^15^, but the overall *Synechococcus* contribution to phytoplankton composition was small. Larger phytoplankton were rare and the ratio of photo-to-heterotrophic plankton biomass was nearly 1:1 throughout the transect. A linear regression model showed no significant explanatory power of relative biomass composition for POM concentrations and elemental ratios (Supplementary Table 3, Supplementary Fig. 4). Instead, POM variation was explained equally well by a combination of a sinusoidal diel plus either temperature or nutricline depth model (Supplementary Fig. 5, Supplementary Fig. 6, Supplementary Table 4, Supplementary Table 5). The models lent support for both the translation-compensation and nutrient supply hypotheses. As such, we were statistically unable to distinguish between these two ecological stoichiometry hypotheses based solely on our Indian Ocean data.

**Fig. 5:**
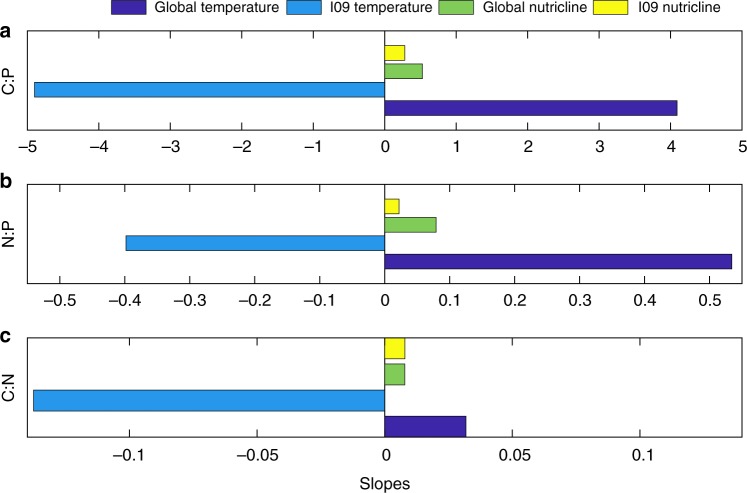
Global and Indian Ocean environmental correlations. Mean of slope estimates for global and Indian Ocean (IO9N) transect particulate organic matter (POM) concentrations and ratios. The sign of the slope indicates the average relationship between the POM concentration (ratio) and the environmental parameter (temperature or nutricline depth). Slopes are fitted to a linear regression model (see Methods)

To further understand how POM stoichiometry was regulated, we next compared the observed relationships for environmental variation and POM composition within the Indian Ocean with previously seen global trends^1^ (Fig. 5, Supplementary Fig. 7). While the nutricline depth was positively related to POM elemental ratios for both the Indian Ocean and globally, the relationship for temperature flipped from negative in the Indian Ocean to globally positive. This suggests that the relationship between temperature and POM stoichiometry is not uniform. We further searched the global C:N:P database for all surface transects with strong temperature and nutricline depth correlations (Supplementary Fig. 8). This analysis confirmed the observations in the Indian Ocean, whereby the correlation between nutricline depth and POM stoichiometry was consistently positive. In contrast, the correlation between temperature and POM stoichiometry could be both positive and negative, leading us to reject the translation-compensation hypothesis. It is worth noting that all these cruises were from tropical and subtropical ocean leaving it currently unknown how temperature vs. nutrient supply control higher latitude POM ratios. Nevertheless, the analysis suggests that for at least low latitude regions, nutrient availability is the primary control on POM stoichiometry.

### Proposed model relating N:P supply ratio to gyre POM C:N:P

**Fig. 6:**
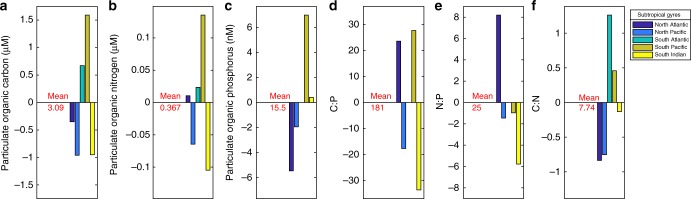
Gyre anomalies for particulate organic matter concentration and ratios. Anomalies are relative to gyre mean (red text) for **a** particulate organic carbon (POC), **b** particulate organic nitrogen (PON), **c** particulate organic phosphorus (POP), **d** POC:POP (C:P), **e** PON:POP (N:P), and **f** POC:PON (C:N). There are no POP, C:P, or N:P data for the South Atlantic gyre

While the POM C:P and N:P ratios in SIO were above Redfield proportions, they were still substantially lower than observed in several other low nutrient ocean regions. To further understand the biogeochemical controls on POM cycling, we compared the POM concentrations and ratios in SIO gyre to the North Atlantic, South Atlantic, North Pacific, and South Pacific subtropical gyres (Fig. 6, Supplementary Fig. 9). The mean gyre concentration for POC, PON, and POP was 3.1 µM, 0.37 µM, and 16 nM respectively and our observed concentrations in the SIO gyre were generally consistent with these levels (Supplementary Table 6). However, there was also a clear difference in the levels and ratios across gyres (Supplementary Table 7, ANOVA *p*-values < 1E−16). The South Indian and North Pacific observations had anomalously low median POM concentrations, the North Atlantic was near the mean, and the South Pacific and South Atlantic gyres had median POM concentrations at or above the means. Median C:P and N:P ratios were near or slightly above Redfield proportions in the SIO gyre (C:P = 147:1, N:P = 19:1), near the average in the North and South Pacific, and elevated in the North Atlantic subtropical gyre (C:P = 205:1, N:P = 33:1) (no POP data for the South Atlantic). Median C:N ratios ranged from 6.9 (North Atlantic gyre) to 9.0 (South Atlantic gyre). The highest C:N ratios were found in the gyres with the highest median POM concentrations. Thus, there were significant differences in POM ratios across gyres (Supplementary Table 7).

**Fig. 7:**
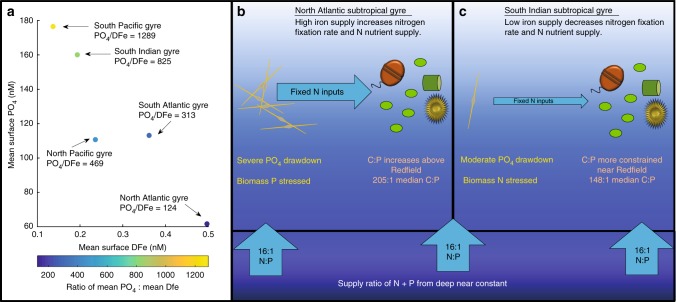
Conceptual model for regulation of ocean gyre biogeochemistry. **a** Nutrient levels across gyres: median surface phosphate to dissolved iron in top 50 m. **b** Iron supply model for C:P in North Atlantic and **c** South Indian Ocean subtropical gyres. Median C:P values are from gyre comparison (North Atlantic, Supplementary Table 6) and this cruise

We hypothesized that low iron (Fe) supply could influence the elemental ratios via Fe-controls on regional N_2_-fixation rates and the relative degree of N vs. P stress (Fig. 7)^20^. In regions with low N_2_-fixation rates, a relatively higher P vs. N availability would lead to lower POM C:P and vice-versa for regions with high rates leading to high POM C:P. Thus, Fe controls on N_2_-fixation may influence the nutrient supply ratio of nitrogen vs. phosphorus which in turn would affect POM C:P^5^. Previously measured dissolved Fe concentrations^11,21^ in subtropical gyres have an inverse relationship with surface phosphate^22^ (Fig. 7a). Here, the South Indian and South Pacific gyres have the highest phosphate concentrations, but lowest dissolved iron concentrations. The lowest PO4:Dfe concentration ratio was found in the North Atlantic gyre. To begin to evaluate this hypothesis, we measured ratios of POM iron to carbon and phosphorus. We detected lower labile particulate iron to POC (LPFe:C) ratios in the SIO gyre (17.8 nM/µM) and typical of a low iron ecosystem (Supplementary Table 1). This was seen for both labile and refractory pFe. In contrast, pFe:C was elevated in the EqIO (22.3 nM/µM) region and further increased into the BoB (44.5 nM/µM) (Supplementary Table 1). Thus, there appeared to be lower iron stress north of EqIO and the highest degree of iron stress in the gyre. As such, the C:P ratio in the SIO compared to the North Atlantic gyre may be depressed due to lower Fe, lower P, and higher N availability (Fig. 7b, c). The regional LPFe:C and LPFe:P mean ratios increased toward the north, further indicating reduced iron stress in the phytoplankton community in EqIO and BoB (Supplementary Table 6). Thus, the elevated iron stress in the South Indian Ocean may suppress C:P in the gyre.

## Discussion

The quantification of POM concentrations in the eastern Indian Ocean allowed us to test current hypotheses for how elemental ratios are regulated as well as identify regional differences in biogeochemical functioning. Our findings directly evaluate three proposed mechanisms (allometric diversity, temperature, and nutrient supply) that may explain deviations in POM stoichiometry. Consistent with past studies^23,24^, *Prochlorococcus* dominated the phytoplankton community and the ratio of photo-to-heterotrophic plankton biomass was nearly 1:1 throughout the transect. Thus, we only observed minor changes in the community structure leading us to reject the allometric diversity hypothesis. However, there are caveats to this conclusion. First, genetic diversity within groups (e.g., ecotypes) may determine growth physiology leading to unique elemental composition^25^. However, no systematic patterns have yet been determined at this level of phylogenetic resolution. Secondly, heterotrophic bacteria did constitute a slightly larger portion of the relative biomass when C:P and C:N ratios were higher. We find this an unlikely driver as heterotrophic bacteria tend to have lower C:nutrient ratios in comparison to phytoplankton due to C limitation^5,^^26^. Thus, there is little support that changes in plankton community composition is the primary control on POM stoichiometry in this region.

The unique environmental conditions in the Indian Ocean lead us to support the nutrient supply hypothesis for low latitude marine ecosystems. For this analysis, we assumed nutricline depth was a proxy for nutrient supply rates to the surface and that a deeper nutricline would be indicative of increased surface nutrient stress. We found that the C:P, N:P, and C:N ratios were highest in the SIO gyre and decreased when the nutricline shoaled around 10–12°S. Northwards of this latitude, the Indian Ocean is subject to monsoonal circulation patterns and fine-scale variation in the elemental ratios corresponded to observed changes in the nutrient supply. All of the ratios remained above Redfield proportions, reflecting oligotrophic surface conditions during the inter-monsoon season. Between 5°S and 5°N, C:P and C:N ratios increased when nutrient concentrations declined below the mixed layer, but the ratios were elevated at the equator where a band of high chlorophyll was present off Sumatra. While the onset of upwelling in the tropical Indian Ocean is consistent, the magnitude is seasonally variable and underlying mechanisms are complex^27–31^. Furthermore, the positive phase of the Indian Ocean Dipole can influence surface circulation as well^15,29^. Historically, upwelling is also observed off Sri Lanka Dome near 5°N, where POM concentrations were the highest and elemental ratios decreased^13,30^. Within the BoB, the elemental ratios flattened out in the stratified Inter-Monsoon gyre until a final increase putatively driven by increased nutrient supply over the continental shelf in northern BoB. Thus, regional differences in the nutrient supply rates indicated by nutricline depth across the eastern Indian Ocean appeared to regulate POM concentrations and ratios.

Two cruise transects in the North Atlantic shared a positive relationship between temperature and nutrient supply and these provide further support of our hypothesis for how POM ratios are regulated. POM elemental ratios were reported as part of a FS Poseidon (Kahler)^32^ cruise (30°W, 18°N–31°N) and a North Atlantic Bloom Experiment (NABE)^33^ cruise (33°N, 21°W–18°N, 30°W). In both of these cruises, nutrient supply rates were the best predictor for POM ratios and the temperature relationship flipped in comparison to global trends (Supplementary Fig. 8). Since macronutrient supply rates are non-limiting in high latitude regions, other factors (e.g., light, temperature, and plankton growth physiology) likely control C:N:P in such biomes^5,^^34^. In support, a recent study demonstrated that the elemental composition of a phytoplankton was highly regulated by the nutrient supply but the optimal composition (i.e., N:P at maximum growth) was temperature dependent^35^. Thus, there could be an interaction leading to a more pronounced temperature effect in high nutrient conditions, but we reject the translation-compensation hypothesis as the primary driver in low latitude regions.

Stoichiometric variation on diel time scales was observed throughout the region. In support, studies of phytoplankton cultures^36–38^ and communities^38–41^ show a peak in the carbon-nutrient ratio towards the end of the photoperiod. A diel range in C:P of 60 and C:N of 2 were found in *Synechococcus* cultures, but barely any variation in the N:P ratio^36^. The peaks are primarily attributed to daytime fixed carbon storage and troughs from exudation and respiration at night^36,42^. The amplitude of C:P and C:N were larger in a culture than observed in the IO9N transect, which may be due to the presence of heterotrophic lineages or detrital material in field samples. The diel cycling of POC accumulation and degradation could also influence nutrient cycling within the whole microbial community. Diel changes in the surface area to volume ratio of phytoplankton can limit their nutrient uptake and the timing of their release of photosynthetically-derived nutrients can directly impact the ambient nutrient concentrations for heterotrophic bacteria. In addition, heterotrophic grazers could compensate for low-quality prey (high C:N, C:P) by increased feeding at night^38^. It was unclear if the N:P ratio residuals displayed a diel cycle leading us to conclude that daily N and P uptake was fairly synchronized in this region. If N fixation played a large role during the IO9N transect, we would expect the N:P ratio to increase during the daytime^43^ but the absence of this trend suggested depressed N-fixation rates. Our results illustrate that the amplitude of daily C:P and C:N peaks is of a comparable magnitude to changes in the ratios across ocean regions, but the lack of N:P cycling indicates a constraint on additional N inputs.

We hypothesized that low Fe supply depresses the elemental ratios via controls on N_2_-fixation rates and the relative degree of N vs. P stress^20^. In contrast, high Fe inputs and increased nitrogen fixation may lead to elevated N and intense P drawdown. We propose that this mechanism leads to divergent C:P and N:P ratios in the North Atlantic Ocean vs. the South Indian Ocean gyre. In the eastern Indian Ocean, the regional LPFe:C and LPFe:P mean ratios increased toward the north, indicating northward positive gradient iron availability for the phytoplankton community (Supplementary Table 1). Higher N-fixation rates in the Arabian Sea than at the equator and SIO gyre along 69°E were attributed to higher dissolved iron in the Arabian Sea^44^. Our depressed C:P ratios in the SIO gyre are consistent with inverse model results and observations of the western SIO gyre^4,40^. Thus, the SIO gyre may represent a low C:P extreme for ocean gyres. As such, the variations in particulate elemental ratios observed in the Indian Ocean are distinctive and impose new constraints on how ocean C:N:P is regulated.

## Methods

### Sample collection and analysis procedures

Seawater samples were collected during the RR1604 GO-SHIP IO9N cruise aboard *R/V Roger Revelle* from March 22–April 24, 2016. Transect coordinates began at 31° 02′01″S/110° 27′28″E off Western Australia and ended at 16° 44′15″N/90° 08′77″E in the BoB (Fig. 1). In total, samples for POC, PON, and POP were taken at 238 stations. Samples for particulate iron were collected from 24 separate trace-metal clean casts off the stern at 20 m depth. Flow cytometry samples for phytoplankton and bacteria cell counts were collected from the mixed layer (~20 m) at 31 GO-SHIP stations. All cruise POM data is available on BCO-DMO (https://www.bco-dmo.org/dataset/734915). Nutrients data for this cruise were provided by Jim Swift/SIO and Susan Becker/SIO and is available at https://cchdo.ucsd.edu^45^.

Water was collected from a circulating seawater system distributed via plastic tubing for POC/PON/POP around 3 m deep. An underway system was chosen to vastly increase sampling coverage, replicate number, and sample volume. The water intake is located near the ship sea chest, which may have missed particle production in the subsurface. The circulating seawater was never turned off during the entirety of the transect and kept at a constant flow. Water was passed through a 30 μm nylon mesh (Small Parts #7050-1220-000-12) to remove larger plankton and particles from the sample. Each replicate was collected into a separate 8.5 L plastic carboys (Thermo Scientific, Waltham, MA). In between stations, carboys were rinsed with 30 μm filtered sample water just prior to collection. Six 8 L seawater samples were divided into POC/PON and POP triplicates. Carboys were placed at ~45° angle to avoid particle settling below the nozzle. Each replicate was passed through a 25 mm pre-combusted (500 °C for 5 h) GF/F filter (Whatman, Florham Park, NJ) with a nominal pore size of 0.7 μm. The vacuum filtration was an in-line setup with 25 mm filter holders connected to an aspirator pump at −0.08 MPa. POP filters were rinsed with 5 mL of 0.17 M Na_2_SO_4_ to remove traces of dissolved phosphorus from the filter. All filters were stored in pre-combusted aluminum packets and immediately frozen at −80 °C during the cruise and −20 °C for shipment.

### Particulate organic carbon/nitrogen

Prior to analysis, the filters for POC and PON were dried according to the JGOFS protocol^46^. The protocol has a detection range of 0.43–43.13 µM for POC and 0.037–7.39 µM for PON in sea water^46^. First, the filters were dried in an incubator at 55 °C for 24–48 h and then stored in a desiccator with concentrated HCl fumes for 24 h to remove inorganic carbonates. Secondly, the filters were dried again at 55 °C for 48 h before being folded and packed into pre-combusted tin capsules (CE Elantech, Lakewood, NJ). The packaged filters are analyzed on a CN FlashEA 1112 Elemental Analyzer (Thermo Scientific, Waltham, MA) against an atropine standard curve (chemical formula C_17_H_23_NO_3_).

### Particulate organic phosphorus

POP was analyzed according to a modified ash-hydrolysis protocol^47^. Thawed filters were placed in along with a corresponding standard curve of KH_2_PO_4_. 2 mL of 0.017 M MgSO_4_ was added to the acid-washed glass vials containing filters and covered with pre-combusted aluminum foil. The vials were placed in an incubator at 90 °C for 24 h and then combusted (500 °C, 2 h). Once cooled, 5 mL 0.2 M HCl was added and incubated at 90 °C for at least 30 min. Next, the supernatant plus 5 mL Milli-Q water was mixed with 2:5:1:2 parts ammonium molybdate tetrahydrate, 5 N sulfuric acid, potassium antimonyl tartrate, and ascorbic acid for 30 min. Finally, the standards and samples were analyzed on a spectrophotometer at a wavelength of 885 nm to determine POP concentration with an assay detection limit 0.1 nmol L^−1^.

### C:N, C:P, and N:P ratios

Each filter analyzed for both POC and PON was treated as a replicate with a corresponding POC/PON ratio. The ratios of POC/POP and PON/POP were taken from the mean concentrations. The standard deviation for C:P and N:P was calculated as a pooled sample:

1 $$\sigma _{\mathrm{CN}} = \surd \left( \left( \Sigma \left( {\mathrm{CN}}_{i} - {\mathrm{CN}}_{\mathrm{ave}}^{2} \right) \right)/n \right),$$

2 $$\sigma_{\mathrm{NP}} = {\mathrm{N}}_{\mathrm{ave}}/{\mathrm{P}}_{\mathrm{ave}} \times \left( \surd \left( \left( \sigma_{\mathrm{N}}/{\mathrm{N}}_{\mathrm{ave}} \right)^{2} + \left( \sigma_{\mathrm{P}}/{\mathrm{P}}_{\mathrm{ave}} \right)^{2} \right) \right),$$

3 $$\sigma _{\mathrm{CP}} = {\mathrm{C}}_{\mathrm{ave}}/{\mathrm{P}}_{\mathrm{ave}} \times \left( \surd \left( \left( \sigma _{\mathrm{C}}/{\mathrm{C}}_{\mathrm{ave}} \right)^{2} + \left( \sigma_{\mathrm{P}}/{\mathrm{P}}_{\mathrm{ave}} \right)^{2} \right) \right),$$

### Relative biomass estimates

Samples for biomass were collected directly into 2 mL cryovials from Niskin bottles at sea, and fixed with freshly made and 0.2 µm filtered paraformaldehyde. After fixation for 1 h at 4 °C in the dark, samples were frozen at −80 °C until analysis. Cell counts were run on a BD FACSJazz flow cytometer equipped a 200 mW 488 nm laser. *Prochlorococcus* was determined by forward scatter and red fluorescence, and *Synechococcus* distinguished by emission in the green and yellow wavelengths. Small eukaryotes were the autofluorescing cells outside of the cyanobacterial gates. Biomass estimates were based on literature values of carbon per cell based on geometric means of forward scatter for each group^48^. Relative biomass estimates were used in this study.

### Particulate and dissolved Fe

Trace metal samples were collected from 5 L Teflon-coated Niskin-X bottles hung on Kevlar line. Niskin bottles were transferred to a clean bubble immediately after sample collection. Samples for dissolved metal analysis were filtered through acid-washed 0.4 µm polycarbonate filters using a vacuum filtration apparatus and acidified using Optima grade HCl. Particulate samples were collected by filtering directly from pressurized Niskin bottles onto 0.45 µm Supor membranes. All samples were handled and stored using trace metal clean protocols. Dissolved samples were analyzed using an ESI seaFAST SP2 coupled to a Perkin Elmer Nexion 350D ICP-MS. Samples were passed through an ESI preconcentration column and buffered in-line with ammonium acetate buffer. Metals were eluted off the column and analyzed in DRC mode using ammonia gas. Samples were quantified using standard additions; each sample was spiked with two additions averaging roughly 100% and 200% of the sample concentration. Labile particulate metals were leached^49^ and were analyzed using a Thermo Element 2 HR-ICP-MS^50^.

### Statistical model analysis

All analyses were completed in MATLAB. Nutricline depth was chosen as a proxy for nutrient supply, and was determined by a threshold nitrate concentration of 1 µM. Depth profiles of nitrate concentrations were analyzed using an AutoAnalyzer shipboard, run by the SIO HydroLab according to standard methods^51^. Mixed layer depths (MLD), isothermal layer depths, and barrier layer thickness were calculated according to Rao and Sivakumar^52^ (Supplementary Fig. 4). MLD is the depth where the change in potential density anomaly (*σ*_*t*_) equals the surface *σ*_*t*(*z*=0)_ plus a change in 0.5 °C (**Δ***T*) times the thermal expansion coefficient (d*σ*/d*t*).

4 $${\mathrm{Mixed}}\,{\mathrm{layer}}\,{\mathrm{depth}}\,\left( {{\mathrm{MLD}}} \right), \, {\mathrm{where}} \, {{\sigma }}_{{{t}}\left( {{{z = h}}} \right)} = \sigma _{{{t}}\left( {{{z = 0}}} \right)} + {\Delta} {T}\,{{\mathrm{d}\sigma /\mathrm{d}t}},$$

5 $${\mathrm{Isothermal}}\,{\mathrm{layer}}\,{\mathrm{depth}}\,\left( {{\mathrm{ITL}}} \right), \, {\mathrm{where}}\, {{\theta = \theta }}_{{{(z = 0)}}} + {{\Delta {T}}},$$

6 $${\mathrm{Barrier}}\,{\mathrm{layer}}\,{\mathrm{thickness}}\,\left( {{\mathrm{BLT}}} \right) = {\mathrm{ITL}}\,-\,{\mathrm{MLD,}}$$

SST values were from the underway temperatures by the Hydro Lab (HLT) using the following correction^53^:

7 $${\mathrm{SST}}\left( {{\mathrm{estimated}}} \right) = {\mathrm{0}}{\mathrm{.001424}} \ast {\mathrm{HLT}}^{\mathrm{2}} + {\mathrm{0}}{\mathrm{.950053}} \ast {\mathrm{HLT}} + {\mathrm{0}}{\mathrm{.048227,}}$$

Statistical models were fitted using one or two predictor variables (SST (°C), nutricline depth (m), or MLD, as well as time) (Supplementary Table 5). The models were also fitted against all stations south of 5°N to examine the influence from the BoB on the fits (Supplementary Table 6). RMSE and *R*^2^ were used to compare across models. If a daily diel rhythm was identified (Supplementary Table 1, Supplementary Fig. 3), a sine function was added to the model with a fixed period of 24 h.

8 $$\begin{array}{l}y = p\left( 1 \right)_y {\ast} \sin \left( {\frac{{\mathrm{Tot}}\,{\mathrm{Hrs}} {\ast} 2{\mathrm{\pi}}}{{24}} + p\left( 2 \right)_y} \right) + \left( {p\left( 3 \right)_y + \hskip 2ptp\left( 4 \right)_y} \right. \times \left( {\mathrm{SST}},{\mathrm{Znut}},{\mathrm{or}}\,{\mathrm{MLD}} \right){\mathrm{,}}\\ \hskip -105pt {\mathrm{where}}\,y = {\mathrm{POC}},{\mathrm{PON}},{\mathrm{POP}},{\mathrm{C:P}},{\mathrm{C:N}},{\mathrm{or}}\,{\mathrm{N:P}}\end{array}$$

Residuals between the points and 8-point moving average were used for comparing the diel cycles of POM concentrations and ratio at each station. Again, most of the variation could be equally explained by temperature or nutricline depth. Residuals between the points and 8-point moving average were used for comparing the diel cycles of POM concentrations and ratio at each station.

### Global and gyre comparisons

The global observations of concentrations and ratios of POM were from updated POM database^54^. Nitrate concentrations and temperature values were taken at the nearest depth from monthly WOA13 values at 1 km resolution^22,55^. For the gyre comparison, gyre coordinates were determined where the nutricline depth was greater than 150 m for the North Atlantic, South Atlantic, North Pacific, South Pacific, and South Indian Oceans. The latitude blocks for each gyre are as follows: North Atlantic (90°W to 5°W); North Pacific (120°E to 100°W); South Atlantic (60°W to 10°E); South Pacific (60°W to 150°E); South Indian (30°E to 150°E). A map and boxplot of observations from each gyre and the new Indian Ocean values are shown in Supplementary Fig. 7. Average gyre surface phosphate concentrations were taken from 0 m WOA13 values over each gyre area (Fig. 7)^22^. Average gyre surface dissolved iron concentrations were taken from all data point in the top 50 m over each gyre surface area using the more recent Tagliabue et al. database^21^. For the global comparison, POM observations were filtered to only include the top 30 m. Temperature and nutricline values were paired with the observations and normalized to the maximum values. Correlation coefficients and slopes were determined separately for the global database stations and the new Indian Ocean observations (Fig. 5). The slopes were determined from a linear regression using a Monte Carlo Metropolis–Hastings algorithm developed for MatlabStan^56,57^. The scatter plots, linear fits and correlations are shown in Supplementary Fig. 6.

## Electronic supplementary material

Description of Additional Supplementary Files

Supplementary Information

Supplementary Data 1

## Data Availability

Particulate organic matter data that support the findings of this study has been deposited in BCO-DMO as cited: Martiny, Adam and Lomas, Michael (2018) Particulate organic matter (PON, POC, POP) concentrations collected on R/V Roger Revelle cruise RR1604 along the hydrographic line IO9 in the eastern Indian Ocean from March to April 2016. Biological and Chemical Oceanography Data Management Office (BCO-DMO) https://www.bco-dmo.org/dataset/734915. Data for the GO_SHIP line I09N can be found at https://cchdo.ucsd.edu/.
