# Supplementary material for: Nutrient supply controls particulate elemental concentrations and ratios in the low latitude eastern Indian Ocean
Source: Nat Commun. 2018 Nov 19;9:4868. doi: 10.1038/s41467-018-06892-w (PMC6242840; doi:10.1038/s41467-018-06892-w)
Supplement: Supplementary file 1 — Description of Additional Supplementary Files [file 41467_2018_6892_MOESM1_ESM.docx]

**Title:** Supplementary Data 1

**Description:** Contains 238 station observations for particulate organic carbon, nitrogen, and phosphorus. The triplicate measurements are provided, as well as the latitude, longitude, date, and time collected
