# Supplementary material for: Nutrient supply controls particulate elemental concentrations and ratios in the low latitude eastern Indian Ocean
Source: Nat Commun. 2018 Nov 19;9:4868. doi: 10.1038/s41467-018-06892-w (PMC6242840; doi:10.1038/s41467-018-06892-w)
Supplement: Supplementary file 2 — Supplementary Information [file 41467_2018_6892_MOESM2_ESM.pdf]

Supplementary Information for:
Nutrient supply controls particulate elemental concentrations and ratios in the low latitude eastern Indian Ocean.

Catherine A. Garcia[1], Steven E. Baer[2], Nathan S. Garcia[1], Sara Rauschenberg[2], Benjamin S. Twining[2], Michael W. Lomas[2], Adam C. Martiny[1,3,*]

1. Department of Earth System Science, University of California at Irvine, Irvine, CA, 92617, USA

2. Bigelow Laboratory for Ocean Sciences, East Boothbay, ME, 04544, USA

3. Department of Ecology and Evolution, University of California at Irvine, Irvine, CA, 92617, USA

***Corresponding Author:** amartiny@uci.edu

Supplementary Figures

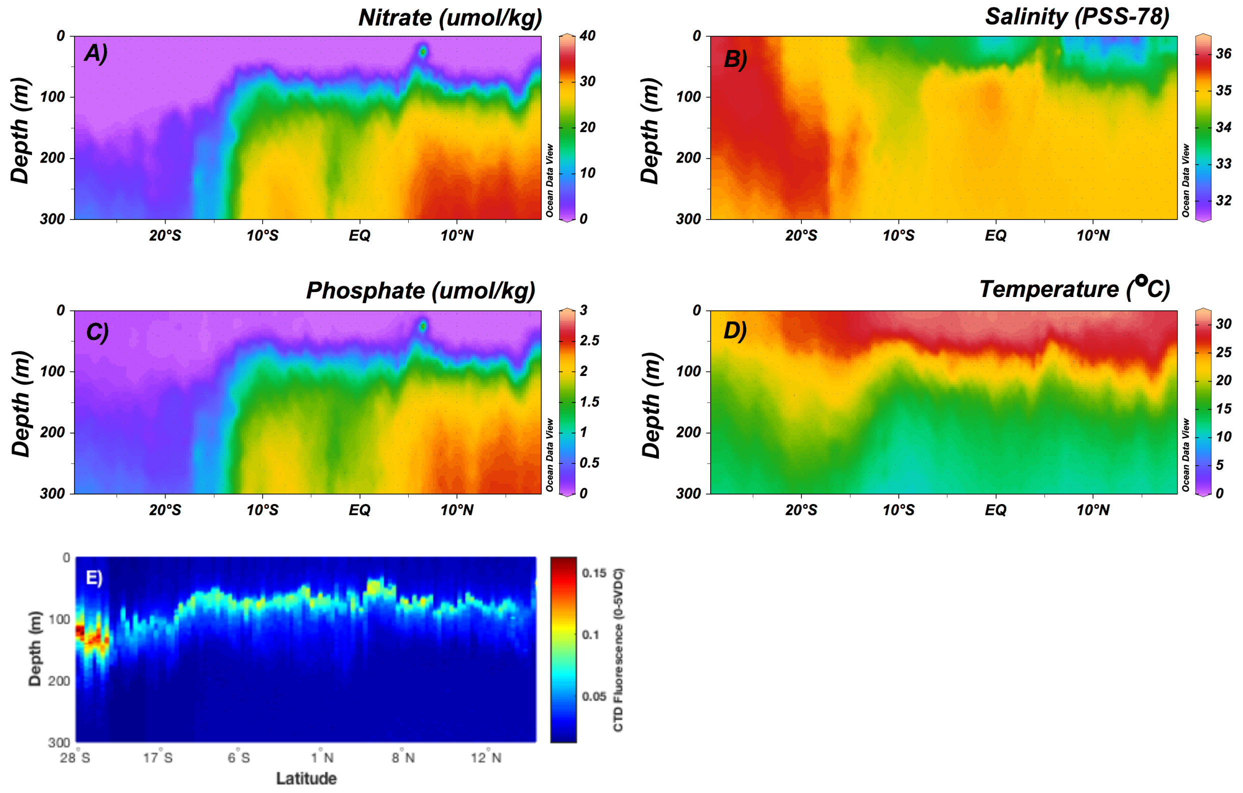

Supplementary Figure 1: I09 GO-SHIP section profiles. From top to bottom: A) Nitrate (μmol/Kg), B) Salinity (PSS-78), C) Phosphate (μmol/Kg), and D) CTD temperature (°C). Images made in Ocean Data View. E) CTD fluorescence profiles mapped in MATLAB.

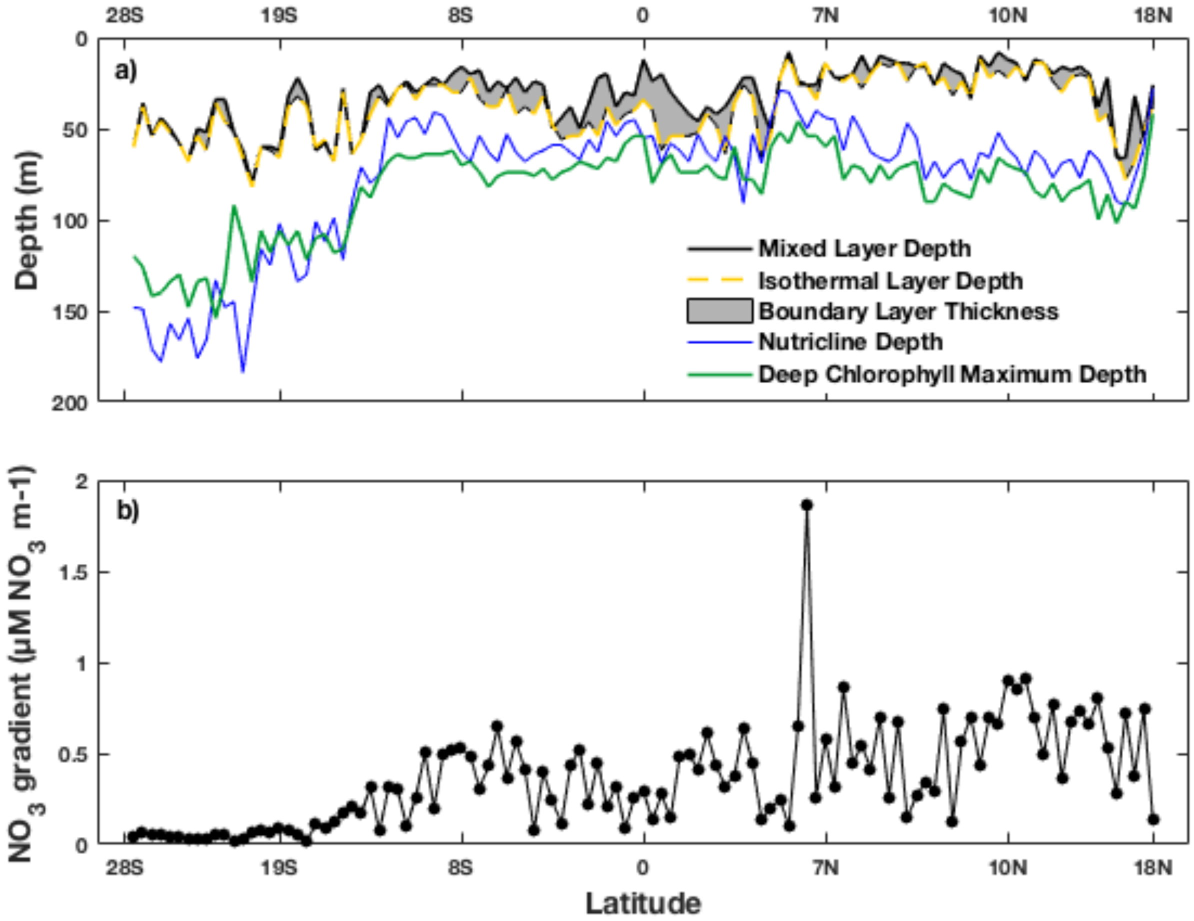

Supplementary Figure 2: Surface layer depths and gradients. a) Mixed layer depth (black line), nutricline depth (blue line), isothermal layer (ITL) dashed red line, and the deep chlorophyll maximum (DCM) is the green line. The barrier layer thickness is shaded in grey between the MLD and ITL and thickest near the equator. Because nutricline and DCM depths were deeper than mixed layer depths, it is likely that biological uptake influenced nutricline depths in the SIO gyre and Bay of Bengal. b) NO_3 gradient calculated as change in concentration 10m below nutricline depth.

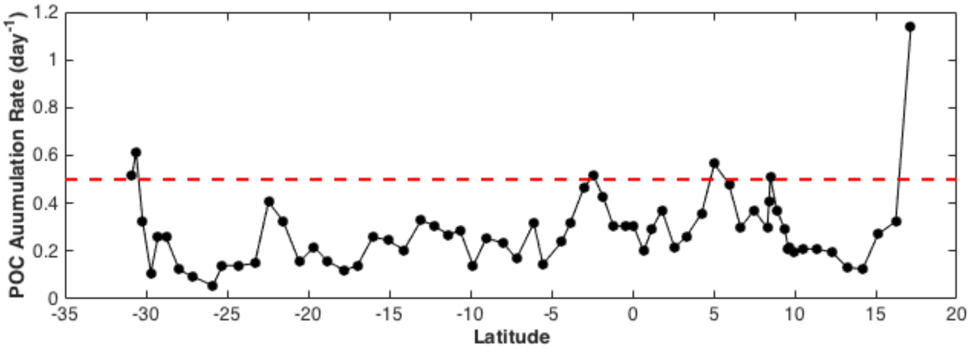

Supplementary Figure 3: Daily accumulation rate of particulate organic carbon (POC). This is the peak to trough difference in daily POC divided by the minimum POC. Red dashed reference line is added at 0.5 day^{-1}.

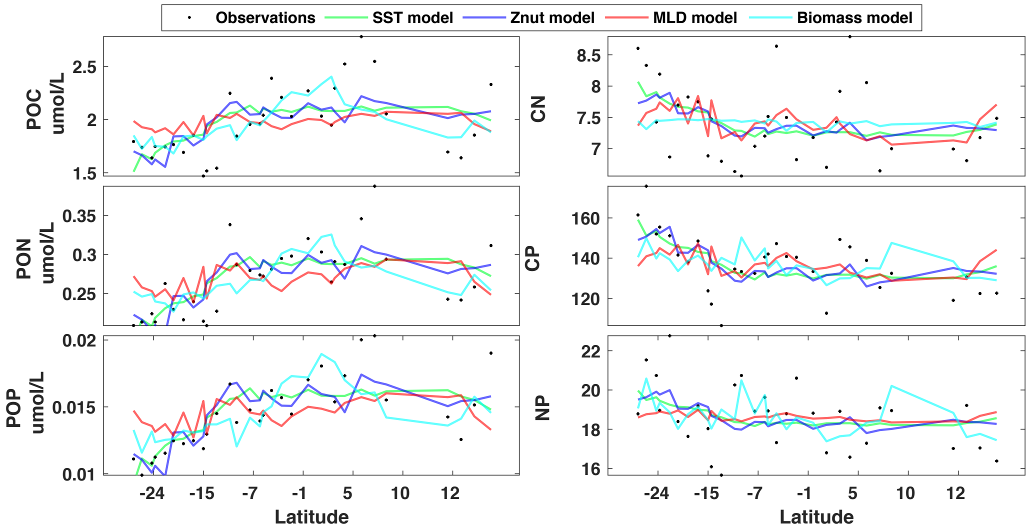

Supplementary Figure 4: Linear Model β0 + β1*x1 + β2*x2 + β3*x3 + β4*x4. Lines are plotted predictions of POM concentrations and ratios at Biomass Stations for f(SST) -green, f(Znut) -blue, f(MLD)-red, f(bact,por,syn,euks)-cyan. The observations are ins black. SST = sea surface temperature. Znut = nutricline depth. MLD = mixed layer depth.

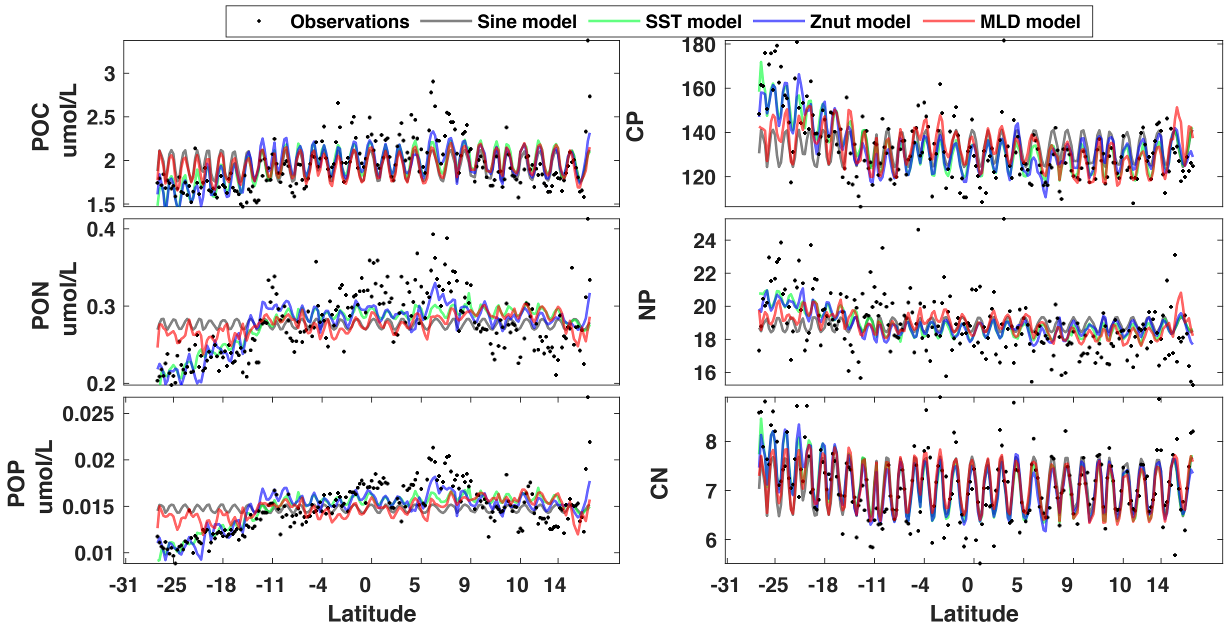

Supplementary Figure 5: Nonlinear sine models with Bay of Bengal. A) Models for POM concentrations and ratios are described in methods. Observations (black circles) and model prediction (Sine-grey, SST-green, Znut-blue, MLD-red) for POC, POP, PON, C:P, N:P and C:N. B) The same except the Bay of Bengal stations above 5N are removed. SST = sea surface temperature. Znut = nutricline depth. MLD = mixed layer depth.

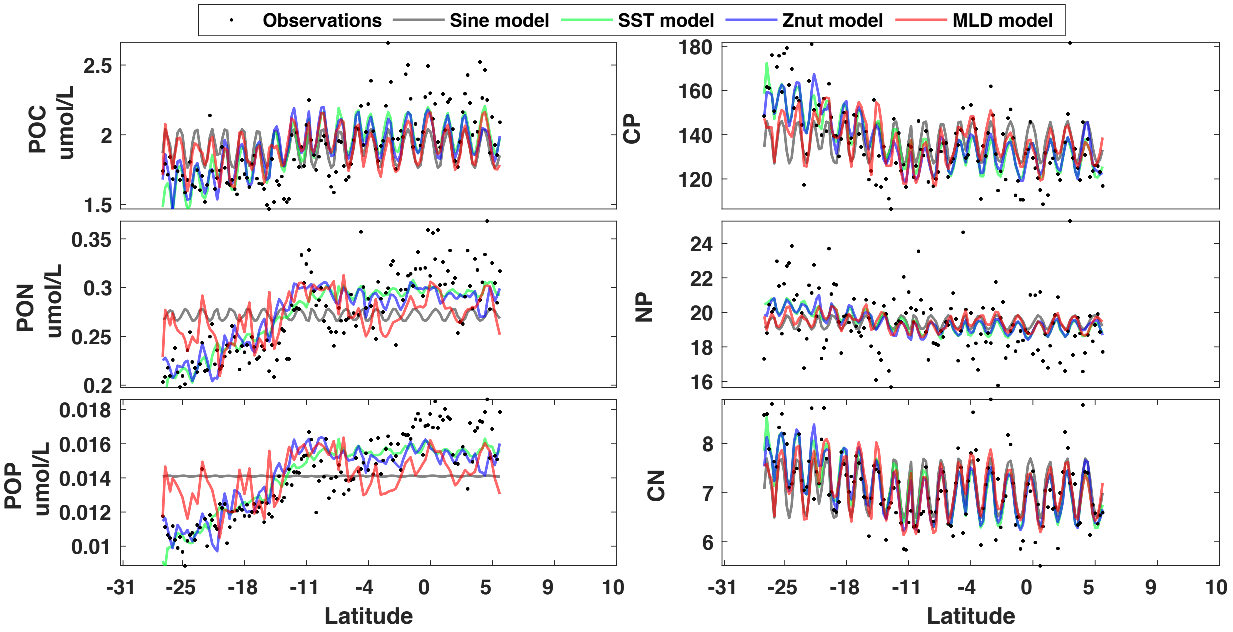

Supplementary Figure 6: Nonlinear sine models without Bay of Bengal (<5N). A) Models for POM concentrations and ratios are described in methods. Observations (black circles) and model prediction (Sine-grey, SST-green, Znut-blue, MLD-red) for POC, POP, PON, C:P, N:P and C:N. B) The same except the Bay of Bengal stations above 5N are removed. SST = sea surface temperature. Znut = nutricline depth. MLD = mixed layer depth.

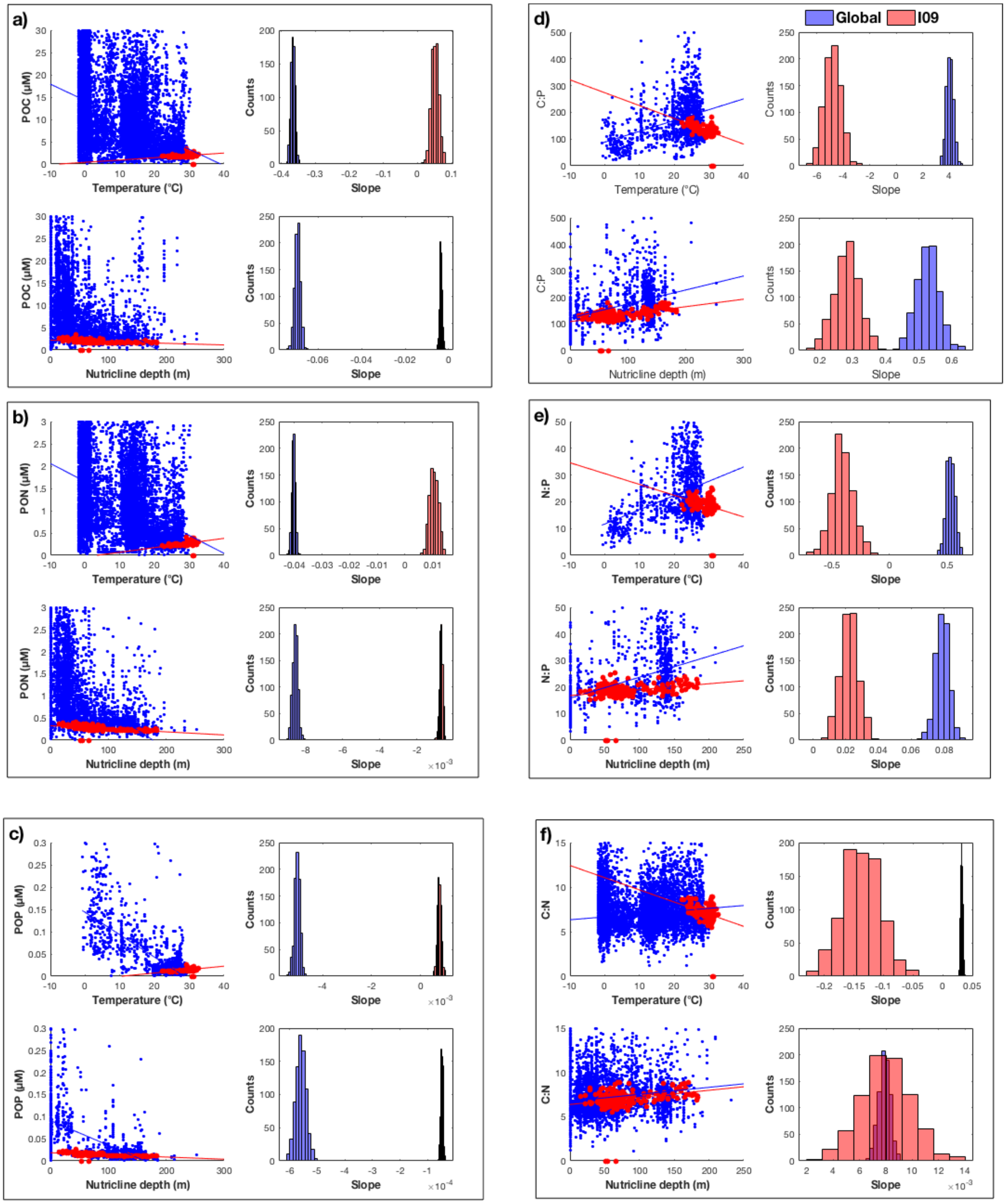

Supplementary Figure 7: Comparison of global and IO9 POM concentrations and ratios to nutricline depth @ 1µM NO_3 and ocean temperature. The slopes are estimates at each station across 1000 iterations, and then averaged across each iteration for the histogram plots (total counts = 1000). The global observations and histogram slopes are in blue. The Indian Ocean observations are in red.

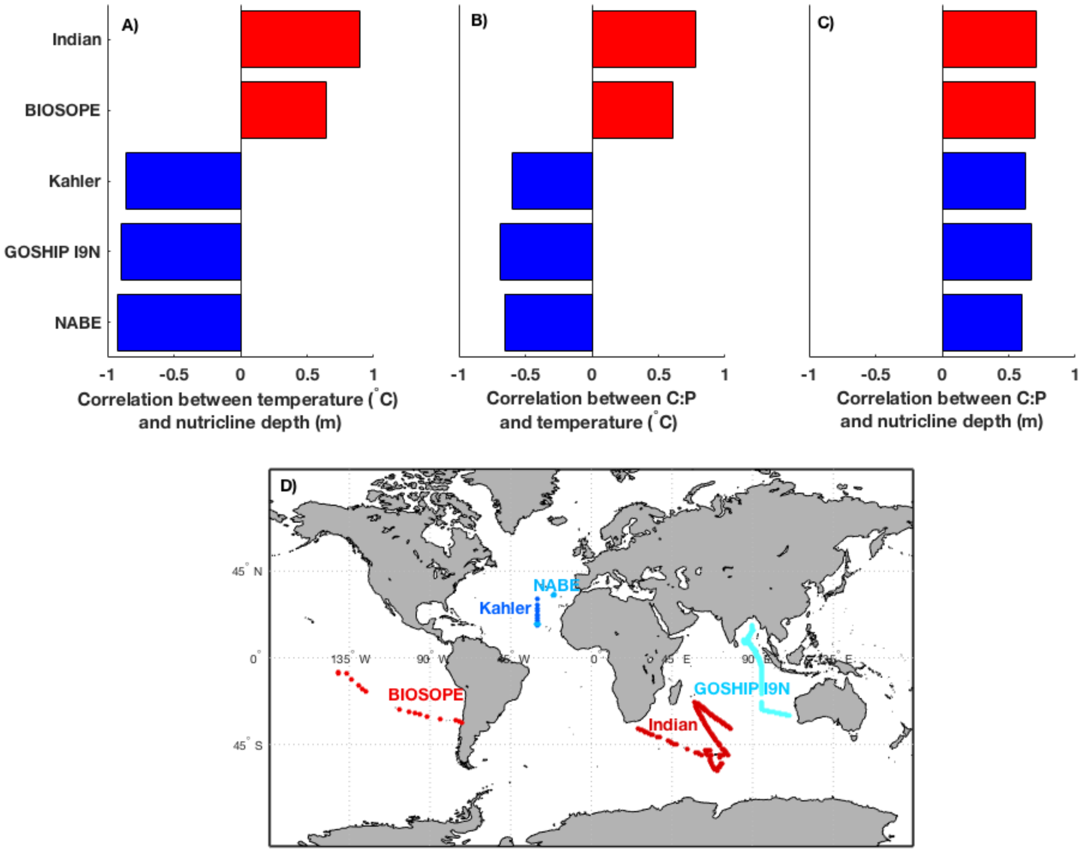

Supplementary Figure 8: Correlation between temperature and nutricline depth among cruise transects with POC:POP (C:P) data, are shown for A) the correlation between temperature and nutricline depth, B) the correlation between particulate C:P ratio and temperature and C) the correlation between particulate C:P ratio and nutricline depth. This study eastern Indian Ocean transect is shown in cyan. Blue bars indicate negative correlations between temperature and nutricline depth of R < -0.5. Red bars indicate positive correlations between temperature and nutricline depth of R > 0.5. Y-axis shows cruise transects for global C:N:P database [1]. D) Map of station locations.

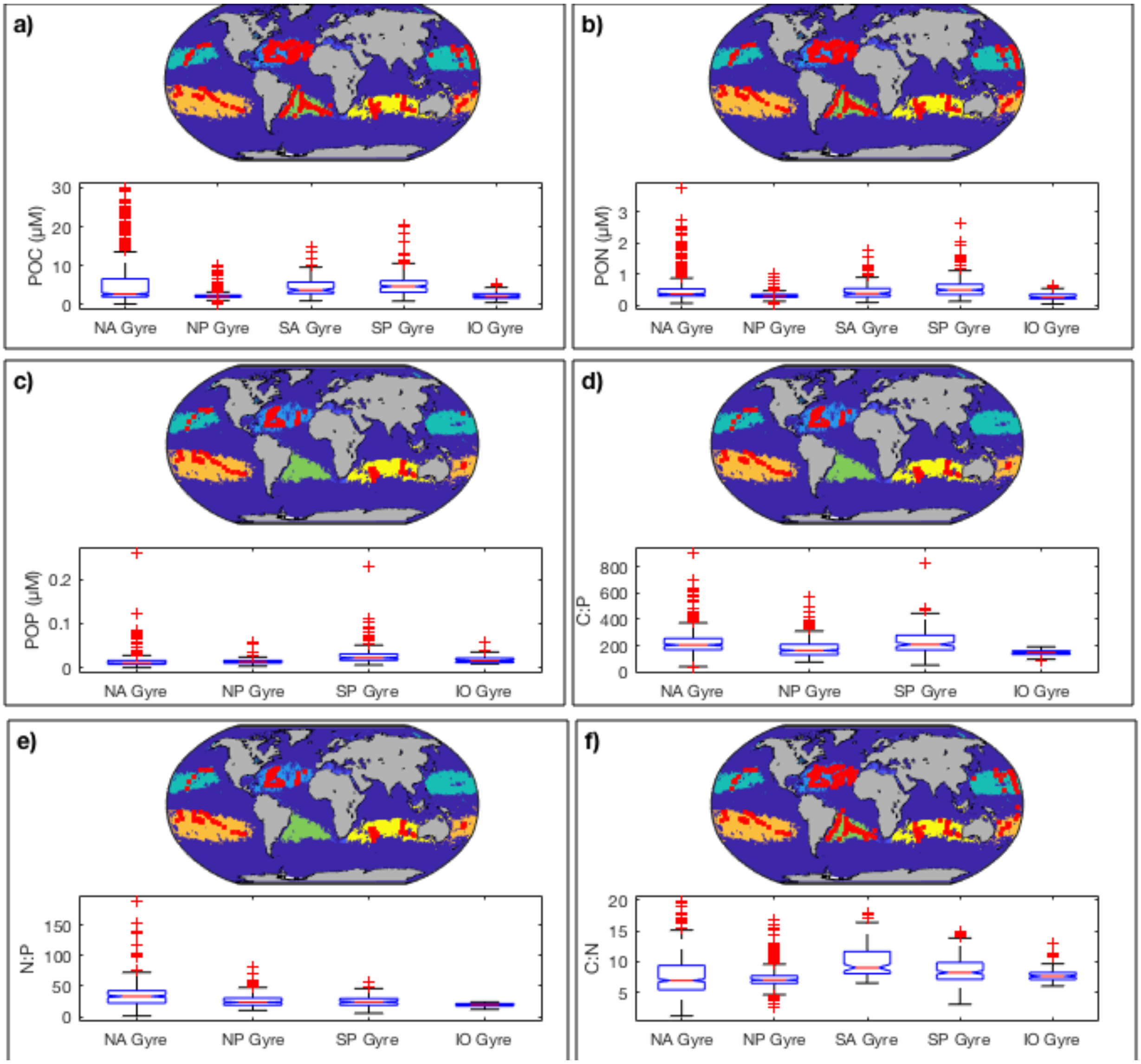

Supplementary Figure 9: Gyre comparison of POM concentrations and ratios. Observations are included in gyres where nutricline depths at 5µM NO_3 are above 150m.

Supplementary Table 1. Regional and transect averages for environmental parameters				
Region	**Indian Ocean Average**	**SIO Gyre**	**EqIO**	**Bay of Bengal**
Latitude	31°S to 19°N	31°S to 12°S	10°S to 5°N	5°N to 19°N
SST (C)	29.10 ± 2.91	25.33 ± 2.56	30.74 ± 0.26	30.79 ± 0.69
Nutricline (m)	78.1 ± 36.9	128.6 ± 36.7	61.6 ± 10.4	61.4 ± 17.0
Mixed Layer (m)	38.0 ± 16.3	53.7 ± 12.5	40.2± 9.4	26.5 ± 14.7
% *Prochlorococcus* biomass	51.8 ± 5.0%	53.9 ± 4.5%	48.6 ± 3.1%	54.9 ± 5.5%
% *Synechococcus* biomass	38.3±3.8%	36.6±2.9%	41.6±2.6%	35.1±2.4%
% Eukaryotes biomass	2.1± 2.0%	0.5± 0.2%	3.6± 2.3%	2.8± 1.2%
% Heterotrophic Bacteria biomass	7.7± 3.9%	9.0± 3.8%	6.2± 2.9%	7.1± 4.3%
Particulate organic carbon (µM)	1.97 ± 0.10	1.84 ± 0.12	2.05 ± 0.10	2.01 ± 0.08
Particulate organic nitrogen (µM)	0.28 ± 0.02	0.25 ± 0.03	0.30 ± 0.02	0.28 ± 0.02
Particulate organic phosphorus (nM)	14.70 ± 0.85	12.27 ± 0.90	15.71 ± 0.81	15.80 ± 0.78
C:P	135.30 ± 11.99	150.51 ± 15.87	131.14 ± 10.38	126.93 ± 8.79
N:P	19.00 ± 2.12	20.14 ± 2.82	19.00 ± 1.97	17.92 ± 1.44
C:N	7.17 ± 0.56	7.55 ± 0.85	6.96 ± 0.52	7.11 ± 0.34
Dissolved iron (nM)	0.15 ± 0.11	0.15 ± 0.05	0.08 ± 0.04	0.26 ± 0.14
Labile particulate iron (pM)	53.6 ± 30.9	32 ± 4	47 ± 17	89 ± 26
LPFe(nM): C(µM) (ratio of means)	26.5	17.8	22.3	44.5
LPFe (pM): P(nM) (ratio of means)	3.6	2.6	3	5.6
POC 24-hr residual amplitude (µM)	0.116	0.037	0.174	0.132
Local time of POC peak min (max)	7:00 (19:00)	5:00 (17:00)	7:00 (19:00)	8:00 (20:00)
PON 24-hr residual amplitude (µM)	5.9 x 10-3	5.5 x 10-3	7.9 x 10-3	9.1 x 10-3
Local time of peak min (max)	13:00 (01:00)	19:00 (07:00)	12:00 (24:00)	13:00 (01:00)
POP 24-hr residual amplitude (µM)	3.8 x 10-4	4.8 x 10-4	3.6 x 10-4	5.9 x 10-4
Local time of peak min (max)	8:00 (20:00)	4:00 (16:00)	7:00 (19:00)	8:00 (20:00)
C:P 24-hr residual amplitude	6.7	2.58	8.80	7.11
Local time of peak min (max)	7:00 (19:00)	8:00 (20:00)	6:00 (18:00)	7:00 (19:00)
N:P 24-hr residual amplitude	0.32	0.47	0.28	0.36
Local time of peak min (max)	17:00 (05:00)	18:00 (06:00)	17:00 (05:00)	18:00 (06:00)
C:N 24-hr residual amplitude	0.29	0.17	0.26	0.37
Local time of peak min (max)	6:00 (18:00)	4:00 (16:00)	6:00 (18:00)	7:00 (19:00)

Supplementary Table 1. Temperature, nutricline depth, mixed layer depth, percent relative biomass, particulate organic matter (POM) concentrations and ratios, dissolved iron, and labile particulate iron. Estimated amplitudes, peak minimum (min) local time and maximum (max) local time from fitted sine functions (see Figure 3) are shown for particulate organic carbon (POC), particulate organic nitrogen (PON), particulate organic phosphorus (POP), POC:POP, PON:POP, and POC:PON. SIO = Southern Indian Ocean, EqIO = Equatorial Indian Ocean.

Supplementary Table 2: One-way ANOVA results Regional Differences					
**SST ANOVA**	SS	df	MS	F	Prob>F
Regions	1436.2	2	718.1	326.3	**<1E-16**
Error	497.3	226	2.2		
Total	1933.5	228			
**Nutricline ANOVA**	SS	df	MS	F	Prob>F
Regions	185406.9	2	92703.5	183.6	**<1E-16**
Error	105503.6	209	504.8		
Total	290910.5	211			
**Mixed Layer ANOVA**	SS	df	MS	F	Prob>F
Regions	25187.3	2	12593.7	81.0	**<1E-16**
Error	32481.7	209	155.4		
Total	57669.0	211			
**POC ANOVA**	SS	df	MS	F	Prob>F
Regions	1.9	2	0.9	10.0	**6.8E-05**
Error	20.7	223	0.1		
Total	22.5	225			
**PON ANOVA**	SS	df	MS	F	Prob>F
Regions	0.1	2	0.0	30.7	**1.7E-12**
Error	0.4	223	0.0		
Total	0.5	225			
**POP ANOVA**	SS	df	MS	F	Prob>F
Regions	0.0	2	0.0	65.1	**<1E-16**
Error	0.0	224	0.0		
Total	0.0	226			
**POC:POP ANOVA**	SS	df	MS	F	Prob>F
Regions	23223.3	2	11611.6	57.3	**<1E-16**
Error	45025.0	222	202.8		
Total	68248.2	224			
**PON:POP ANOVA**	SS	df	MS	F	Prob>F
Regions	186.1	2	93.1	31.4	**9.9E-13**
Error	658.2	222	3.0		
Total	844.4	224			
**POC:PON ANOVA**	SS	df	MS	F	Prob>F
Regions	13.2	2	6.6	15.3	**5.8E-07**
Error	95.7	223	0.4		
Total	108.9	225			

Supplementary Table 2: One-way ANOVA results for POM concentrations, ratios and environmental parameters. Regions are defined as the Southern Indian Ocean Gyre (31°S to 12°S), Equatorial Upwelling (10°S to 5°N), and the Bay of Bengal (5°S to 20°N). SST = sea surface temperature, POC = particulate organic carbon, PON = particulate organic nitrogen, POP = particulate organic phosphorus, and POM = particulate organic matter. Sum of squares (SS), degree of freedom (df), mean squares (MS=SS/df), ratio of mean squared errors, F=MS(Regions)/MS(Error)

Supplementary Table 3: Linear Model Coefficients and Statistics												
Ratio	LinModel	β0	β1	β2	β3	β4	N	DF	Fstat	pVal	R2	RMSE
C:P	f(SST)	232.0	-3.3	0.0	0.0	0.0	30	28	12.8	**0.001**	0.31	12.64
C:P	f(Znut)	120.3	0.2	0.0	0.0	0.0	30	28	13.4	**0.001**	0.32	12.54
C:P	f(MLD)	119.8	0.4	0.0	0.0	0.0	30	28	4.4	**0.045**	0.14	14.18
C:P	f(b,pro,syn,euks)	123.1	154.3	109.5	284.1	-142.4	30	25	1.2	0.337	0.16	14.79
N:P	f(SST)	24.4	-0.2	0.0	0.0	0.0	30	28	2.9	0.101	0.09	1.62
N:P	f(Znut)	17.4	0.0	0.0	0.0	0.0	30	28	4.6	**0.041**	0.14	1.58
N:P	f(MLD)	18.0	0.0	0.0	0.0	0.0	30	28	0.4	0.551	0.01	1.69
N:P	f(bact,pro,syn,euks)	16.5	17.0	14.1	36.4	-15.4	30	25	2.1	0.109	0.25	1.56
C:N	f(SST)	10.2	-0.1	0.0	0.0	0.0	31	29	5.1	**0.032**	0.15	0.59
C:N	f(Znut)	7.0	0.0	0.0	0.0	0.0	31	29	3.8	0.061	0.12	0.61
C:N	f(MLD)	6.7	0.0	0.0	0.0	0.0	31	29	4.2	**0.048**	0.13	0.60
C:N	f(bact,pro,syn,euks)	7.5	4.4	3.4	4.0	-4.4	31	26	0.0	0.996	0.01	0.68

Supplementary Table 3: Linear Model β0 + β1*x1 + β2*x2 + β3*x3 + β4*x4. Observations (n = 30/31) limited to stations with biomass estimates. SST = sea surface temperature (^{o}C), Znut = nutricline depth (m), MLD = mixed layer depth(m), bact = Heterotrophic bacteria (ugC/L), pro = *Prochlorococcus* (ugC/L), syn = *Synechococcus* (ugC/L), euks = Eukaryotes (ugC/L). All biomass cells pre-filtered through 20µm mesh. All POM concentrations used for C:N:P ratios pre-filtered through 30µm mesh. ANOVA results are for linear model against constant model.

Supplementary Table 4: Models fits for POM concentrations and ratios								
**Ratio/POM**	NonlinModel	p(1)	p(2)	p(3)	p(4)	N obs	R^2	RMSE
**POC (µM)**	f(hour)	-0.18	6.07	1.94	0.00	216	0.19	0.263
**POC (µM)**	f(hours,SST)	-0.18	6.12	0.17	0.06	216	0.39	0.230
**POC (µM)**	f(hours,Znut)	-0.18	6.10	2.25	0.00	216	**0.44**	**0.219**
**POC (µM)**	f(hours,MLD)	0.18	2.94	2.07	0.00	216	0.23	0.258
**PON (µM)**	f(hour)	0.01	1.75	0.28	0.00	216	0.01	0.042
**PON (µM)**	f(hours,SST)	0.01	1.78	-0.07	0.01	216	0.37	0.034
**PON (µM)**	f(hours,Znut)	0.01	1.83	0.34	0.00	216	**0.46**	**0.031**
**PON (µM)**	f(hours,MLD)	0.01	1.69	0.31	0.00	216	0.10	0.040
**POP (µM)**	f(hour)	0.00	5.89	0.01	0.00	217	0.01	2.6E-03
**POP (µM)**	f(hours,SST)	0.00	6.17	-0.01	0.00	217	0.46	1.9E-03
**POP (µM)**	f(hours,Znut)	0.00	2.91	0.02	0.00	217	**0.54**	**1.8E-03**
**POP (µM)**	f(hours,MLD)	0.00	2.74	0.02	0.00	217	0.15	2.4E-03
**C:P**	f(hour)	8.42	3.01	132.78	0.00	215	0.16	13.859
**C:P**	f(hours,SST)	-8.60	6.07	256.91	-4.19	215	**0.52**	10.475
**C:P**	f(hours,Znut)	-8.38	6.11	113.64	0.24	215	**0.52**	10.474
**C:P**	f(hours,MLD)	8.68	2.99	117.77	0.39	215	0.33	12.337
**N:P**	f(hour)	0.46	0.53	18.89	0.00	215	0.03	1.756
**N:P**	f(hours,SST)	0.48	0.63	28.08	-0.31	215	**0.18**	**1.625**
**N:P**	f(hours,Znut)	0.48	0.57	17.55	0.02	215	**0.16**	**1.641**
**N:P**	f(hours,MLD)	0.45	0.58	17.57	0.03	215	0.13	1.669
**C:N**	f(hour)	-0.61	0.05	7.08	0.00	216	0.40	0.522
**C:N**	f(hours,SST)	-0.61	0.02	10.21	-0.11	216	**0.52**	**0.470**
**C:N**	f(hours,Znut)	-0.60	0.04	6.58	0.01	216	**0.53**	**0.465**
**C:N**	f(hours,MLD)	-0.61	0.04	6.75	0.01	216	0.45	0.504

Supplementary Table 4: Models fits for POM concentrations and ratios are described in methods. SST = sea surface temperature (^{o}C), Znut = nutricline depth (m), MLD = mixed layer depth(m).

**Supplementary Table 4: Models fits without Bay of Bengal**								
**Ratio/POM**	NonlinModel	p(1)	p(2)	p(3)	p(4)	N obs	R^2	RMSE
**POC (µM)**	f(hour)	-0.14	6.18	1.90	0.00	137	0.15	0.239
**POC (µM)**	f(hours,SST)	-0.15	6.23	0.04	0.06	137	**0.51**	**0.182**
**POC (µM)**	f(hours,Znut)	-0.15	6.21	2.19	0.00	137	0.42	0.198
**POC (µM)**	f(hours,MLD)	-0.14	6.21	2.20	-0.01	137	0.26	0.223
**PON (µM)**	f(hour)	0.01	0.88	0.27	0.00	137	0.01	0.042
**PON (µM)**	f(hours,SST)	0.00	0.99	-0.11	0.01	137	**0.60**	**0.027**
**PON (µM)**	f(hours,Znut)	0.00	1.45	0.34	0.00	137	0.53	0.029
**PON (µM)**	f(hours,MLD)	0.00	0.88	0.35	0.00	137	0.26	0.036
**POP (µM)**	f(hour)	0.00	1.76	0.01	0.00	136	0.00	2.4E-03
**POP (µM)**	f(hours,SST)	0.00	0.65	-0.01	0.00	136	**0.71**	**1.3E-03**
**POP (µM)**	f(hours,Znut)	0.00	0.19	0.02	0.00	136	0.63	1.4E-03
**POP (µM)**	f(hours,MLD)	0.00	0.51	0.02	0.00	136	0.30	2.0E-03
**C:P**	f(hour)	9.58	3.04	136.59	0.00	136	0.17	15.377
**C:P**	f(hours,SST)	9.15	2.97	253.19	-4.03	136	**0.50**	**11.924**
**C:P**	f(hours,Znut)	8.51	2.98	115.53	0.24	136	**0.52**	**11.719**
**C:P**	f(hours,MLD)	9.36	3.00	112.44	0.53	136	0.34	13.660
**N:P**	f(hour)	0.41	0.78	19.42	0.00	136	0.03	1.779
**N:P**	f(hours,SST)	0.45	0.78	25.67	-0.22	136	**0.11**	**1.708**
**N:P**	f(hours,Znut)	0.47	0.72	18.34	0.01	136	**0.11**	**1.713**
**N:P**	f(hours,MLD)	0.43	0.79	18.31	0.02	136	0.06	1.754
**C:N**	f(hour)	-0.61	0.10	7.09	0.00	137	0.35	0.593
**C:N**	f(hours,SST)	-0.59	0.08	10.82	-0.13	137	**0.53**	**0.505**
**C:N**	f(hours,Znut)	0.57	3.24	6.40	0.01	137	**0.55**	**0.495**
**C:N**	f(hours,MLD)	-0.60	0.08	6.21	0.02	137	0.47	0.535

Supplementary Table 5: Bay of Bengal stations not included. Models fits for POM concentrations and ratios are described in methods. SST = sea surface temperature (^{o}C), Znut = nutricline depth (m), MLD = mixed layer depth(m).

Supplementary Table 6: Median gyre POM concentrations and ratios						
	North Atlantic Gyre	North Pacific Gyre	South Atlantic Gyre	South Pacific Gyre	South Indian Gyre	Gyre Mean
POC (μM)	2.7	2.1	3.8	4.6	2.1	3.07
PON (μM)	0.377	0.302	0.390	0.448	0.261	0.356
POP (μM)	0.010	0.014	-	0.031	0.016	0.018
POC:POP	205.0	163.7	-	177.5	147.7	173.5
PON:POP	33.2	23.6	-	18.9	19.3	23.7
POC:PON	6.9	7.0	9.0	8.1	7.6	7.7

Supplementary Table 6: Median POM concentrations and ratios from surface observations. No POP, C:P, and N:P data points are available for the South Atlantic gyre.

Supplementary Table 7: One-way ANOVA for gyre regions					
**POC ANOVA**	SS	df	MS	F	Prob>F
Regions	4432.1	4	1108.0	52.5	**<1E-16**
Error	35449.5	1680	22.1		
Total	39881.6	1684			
**PON ANOVA**	SS	df	MS	F	Prob>F
Regions	19.7	4	4.9	46.5	**<1E-16**
Error	166.7	1576	0.1		
Total	186.4	1580			
**POP ANOVA**	SS	df	MS	F	Prob>F
Regions	3.1E-2	3	1.0E-2	47.7	**<1E-16**
Error	2.5E-1	1149	2.2E-4		
Total	2.8E-1	1152			
**POC:POP ANOVA**	SS	df	MS	F	Prob>F
Regions	764126.7	3	254708.9	30.9	**<1E-16**
Error	7495867.9	910	8237.2		
Total	8259994.5	913			
**PON:POP ANOVA**	SS	df	MS	F	Prob>F
Regions	27331.6	3	9110.5	43.8	**<1E-16**
Error	183332.3	881	208.1		
Total	210663.9	884			
**POC:PON ANOVA**	SS	df	MS	F	Prob>F
Regions	787.5	4	196.8	33.4	**<1E-16**
Error	8985.3	1525	5.9		
Total	9772.7	1529			

Supplementary Table 7: One-way ANOVA results for POM concentrations and ratios. Regions analyzed are the North Atlantic, South Atlantic, North Pacific, South Pacific, and South Indian gyres. POC = particulate organic carbon, PON = particulate organic nitrogen, POP = particulate organic phosphorus, and POM = particulate organic matter. For POC:POP, POP, and PON:POP there are no observations from the South Atlantic. Sum of squares (SS), degree of freedom (df), mean squares (MS=SS/df), ratio of mean squared errors, F=MS(Regions)/MS(Error).

Supplementary References:

1. Martiny, A. C., Vrugt, J. A. & Lomas, M. W. Concentrations and ratios of particulate organic carbon, nitrogen, and phosphorus in the global ocean. *Sci. Data* **1,** 140048 (2014).
